# Loss‐of‐Function of p21‐Activated Kinase 2 Links BMP Signaling to Neural Tube Patterning Defects

**DOI:** 10.1002/advs.202204018

**Published:** 2022-12-11

**Authors:** Yan Wang, Kaifan Zhang, Jin Guo, Shuyan Yang, Xiaohui Shi, Jinrong Pan, Zheng Sun, Jizhen Zou, Yi Li, Yuanyuan Li, Tianda Fan, Wei Song, Fang Cheng, Cheng Zeng, Jinchen Li, Ting Zhang, Zhong Sheng Sun

**Affiliations:** ^1^ Beijing Institutes of Life Science Chinese Academy of Sciences Beijing 100101 China; ^2^ CAS Center for Excellence in Biotic Interactions University of Chinese Academy of Sciences Beijing 100049 China; ^3^ Institute of Genomic Medicine Wenzhou Medical University Wenzhou Zhejiang 325000 China; ^4^ Beijing Municipal Key Laboratory of Child Development and Nutriomics Capital Institute of Pediatrics Beijing 100020 China; ^5^ Bioinformatics Center & National Clinical Research Center for Geriatric Disorders Xiangya Hospital Central South University Changsha Hunan 410078 China; ^6^ State Key Laboratory of Integrated Management of Pest Insects and Rodents Chinese Academy of Sciences Beijing 100101 China

**Keywords:** BMP signaling, dorsolateral hinge points, neural tube defects, PAK2, single‐cell transcriptome

## Abstract

Closure of the neural tube represents a highly complex and coordinated process, the failure of which constitutes common birth defects. The serine/threonine kinase p21‐activated kinase 2 (PAK2) is a critical regulator of cytoskeleton dynamics; however, its role in the neurulation and pathogenesis of neural tube defects (NTDs) remains unclear. Here, the results show that *Pak2* ^−/−^ mouse embryos fail to develop dorsolateral hinge points (DLHPs) and exhibit craniorachischisis, a severe phenotype of NTDs. *Pak2* knockout activates BMP signaling that involves in vertebrate bone formation. Single‐cell transcriptomes reveal abnormal differentiation trajectories and transcriptional events in *Pak2* ^−/−^ mouse embryos during neural tube development. Two nonsynonymous and one recurrent splice‐site mutations in the *PAK2* gene are identified in five human NTD fetuses, which exhibit attenuated *PAK2* expression and upregulated BMP signaling in the brain. Mechanistically, PAK2 regulates Smad9 phosphorylation to inhibit BMP signaling and ultimately induce DLHP formation. Depletion of *pak2a* in zebrafish induces defects in the neural tube, which are partially rescued by the overexpression of wild‐type, but not mutant *PAK2*. The findings demonstrate the conserved role of PAK2 in neurulation in multiple vertebrate species, highlighting the molecular pathogenesis of *PAK2* mutations in NTDs.

## Graphical abstract

p21‐activated kinase 2 (PAK2) inhibits bone morphogenic protein (BMP) signaling to induce dorsolateral hinge point (DLHP) through regulation of Smad9 phosphorylation. *Pak2* ^−/−^ mouse and *pak2a* ^Δ14^ zebrafish fail to close the neural tube. Human neural tube defects (NTDs) carrying *PAK2* mutations exhibit abnormal *PAK2* expression and BMP signaling. *Pak2* knockout induces abnormal differentiation trajectories during neural tube development.

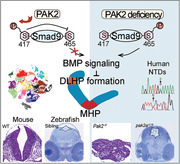

## 1 Introduction

Failure to close the neural tube along the brain (anencephaly and craniorachischisis) and spinal cord (open spina bifida) occurs in approximately 1/1000 births worldwide.^[^ ^1^ ^]^ Neural tube defects (NTDs) are now the second most common type of birth defects after congenital heart defects. Thus, understanding the molecular mechanisms underlying neural tube closure is critical for the prevention of NTDs. In vertebrates, neural tube closure is a highly complex and coordinated process that involves multiple precise cellular events controlled by both genetic and epigenetic factors.^[^ ^2^, ^3^, ^4^ ^]^ Critical processes include the formation of the median hinge point (MHP) and dorsolateral hinge points (DLHPs), around which the neural plates are folded and eventually closed into a neural tube.^[^ ^5^, ^6^ ^]^ In turn, the key molecular regulators of neural tube closure include members of the non‐canonical Wnt/planar cell polarity and sonic hedgehog (Shh)/bone morphogenic protein (BMP) pathways, as well as enzymes involved in folate metabolism.^[^ ^3^ ^]^

The serine/threonine kinase p21‐activated kinase (PAK) 2 functions as the main effector of Rho GTPases and can regulate diverse biological processes via cytoskeletal networks.^[^ ^7^, ^8^, ^9^ ^]^ We previously reported that PAK2 is highly expressed during the fetal period.^[^ ^8^, ^9^ ^]^ Conversely, PAK2 haploinsufficiency is associated with autism‐related behaviors in both mice and humans.^[^ ^8^ ^]^ Notably, we and others have observed that *Pak2* homozygous deletion (*Pak2^−/−^*) was embryonically lethal in mice and the lethality occurred around embryonic day (E) 8.5–10.5, a period in which the neural tube closure occurs.^[^ ^10^ ^]^ This observation suggests that PAK2 has the potential to regulate neural tube formation. However, how PAK2 regulates early neurulation remains unclear.

Emerging evidence has indicated that the secreted protein BMPs contribute to the multifaceted processes of embryogenesis, including the initiation of bone development and the differentiation of different cell types in the central nervous system.^[^ ^11^ ^]^ Notably, a number of BMP members are expressed in the neural plate (BMP4/5) and in the epidermal ectoderm surrounding the neural plate (BMP2/4/5/7) before neural tube closure.^[^ ^12^, ^13^, ^14^, ^15^ ^]^ During neural tube closure, BMP signaling from the surface ectoderm inhibits DLHP formation and negatively regulates dorsal neural tube development.^[^ ^16^, ^17^, ^18^ ^]^ The inhibition of BMP signaling on DLHP formation has been observed in model systems. In mice, *Bmp2* homozygous deletion led to reduced BMP signaling, along with prematurely developed DLHPs and exaggerated bending in the dorsal neural tube.^[^ ^19^ ^]^ In chicks, blockade of BMP signaling through ectopic overexpression of noggin or the dominant‐negative BMPR1 at the ventral midline induces ectopic hinge points, whereas increased BMP signaling abolishes MHP formation and results in the flattened ventral midline.^[^ ^20^ ^]^ Mechanistically, the regulatory Smads (Smad1/5/9) are sequentially phosphorylated following the activation of type I BMP receptors by type II BMP receptors to regulate target gene transcription.^[^ ^21^ ^]^ However, the direct regulatory effects of PAK2 on BMP signaling have not been clarified.

In the study, we examined neural tube morphological alterations in *Pak2^−/−^* embryos, revealing that *Pak2^−/−^* embryos showed a distinct phenotype of craniorachischisis. The molecular changes caused by PAK2 loss‐of‐function were further characterized using bulk embryo RNA‐sequencing (RNA‐seq) and single cell transcriptional sequencing (scRNA‐seq). Moreover, in a large cohort of aborted fetuses with NTDs, we identified two *PAK2* missense mutations and one recurrent splicing mutation in five independent cases, which were associated with decreased *PAK2* expression and enhanced BMP signaling as revealed by NanoString nCounter analysis. Further coimmunoprecipitation (CoIP) assay demonstrated that PAK2 inhibited Smad9 phosphorylation (p‐Smad9) at Ser465 through potentially phosphorylation of Smad9 at Ser417. The effects of *PAK2* mutations on neural tube development were also validated in zebrafish. Together, our results demonstrate a novel function of PAK2 in regulating a key process of neural tube formation, highlighting the deleterious effects of *PAK2* deletion on NTD pathogenesis.

## 2 Results

### 2.1 *Pak2* ^−/−^ Embryos Fail to Develop DLHPs

**Figure 1:**
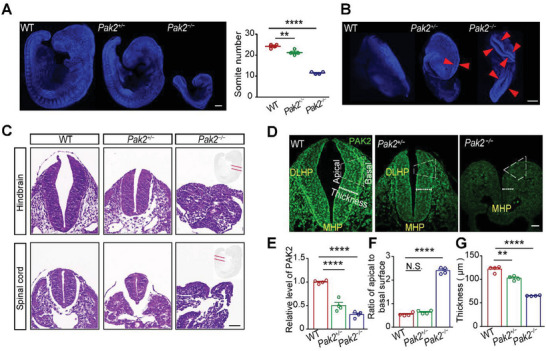
*Pak2^−/−^* embryos fail to develop DLHPs. A) Numbers of somite in WT, *Pak2* ^+/−^, and *Pak2* ^−/−^ embryos at E9.5. (*F* (2, 9) = 188.2, *p* < 0.0001; *p* < 0.0001 for WT versus *Pak2* ^−/−^ mice, *p* = 0.0034 for WT versus *Pak2* ^+/−^ mice). B) *Pak2* ^−/−^ embryos exhibit a characteristic phenotype of craniorachischisis, with an open neural tube (indicated by asterisks) extending from the forebrain to the posterior spinal cord. C) Hematoxylin/eosin‐stained hindbrain and spinal cord of WT and *Pak2* ^−/−^ embryos at E9.5. D) Quantification of the neural tube of WT, *Pak2* ^+/−^, and *Pak2* ^−/−^ embryos at E9.5. E–G)The PAK2 levels (*E*, *F* (2, 9) = 72.68, *p* < 0.0001; *p* < 0.0001 for WT versus *Pak2* ^−/−^ mice, *p* < 0.0001 for WT versus *Pak2* ^+/−^ mice), the ratio of apical to basal surface at the DLHP (*F*, WT, 0.547 ± 0.034; *Pak2* ^−/−^ mice, 2.397 ± 0.067; *Pak2* ^+/−^ mice, 0.640 ± 0.037; *F* (2, 9) = 460.7, *p* < 0.0001; *p* = 0.0001 for WT versus *Pak2* ^−/−^ mice, *p* = 0.335 for WT versus *Pak2* ^+/−^ mice), and the thickness of neural tube (G, *p* = 0.0001 for WT versus *Pak2* ^−/−^ mice, *p* = 0.003 for WT versus *Pak2* ^+/−^ mice) of WT, *Pak2* ^+/−^, and *Pak2* ^−/−^ embryos at E9.5. One‐way ANOVA with Dunnett's multiple comparisons, *n* = 4 embryos for each genotype (A, E, F, G). Scale bar: 200 µm (A,B); 20 µm (C,D).

We found that *Pak2* ^−/−^ mouse embryos failed to survive prenatally, indicating an indispensable role of *Pak2* in normal embryonic development. Upon evaluation of specific embryonic stages by breeding the *Pak2* heterozygous mice, 19 *Pak2* ^−/−^ embryos were identified among 109 embryos at E9.5 (17.4%), decreasing to 3 among 110 embryos at E10.5 (2.7%), indicating that the majority of *Pak2* ^−/−^ embryos failed to survive beyond E10.5. Compared with wild‐type (WT) littermates (24.25 ± 0.479), the somite numbers of *Pak2* ^−/−^ (11.5 ± 0.289) and *Pak2* ^+/−^ (21.25 ± 0.629) embryos displayed a significant decrease at E9.5 (**Figure** **1**A), suggesting that *Pak2* deficiency induces retarded development in mouse embryos. Considering that neural tube closure occurs at a critical period between E8.5 and E10.5 in mice, we examined neural tube morphology and found that *Pak2* ^−/−^ embryos showed a characteristic phenotype of craniorachischisis, with an open neural tube extending from the forebrain to the posterior spinal cord (Figure 1B). Hematoxylin/eosin‐stained histological sections further showed that *Pak2* ^−/−^ embryos failed to elevate the bilateral neural plates at both the hindbrain and spinal cord at E9.5 (Figure 1C).

Specifically, WT embryos developed an MHP and a pair of DLHPs at E9.5 (Figure 1D). The DLHPs then folded to the middle from both sides, resulting in a ratio of apical to basal surface length (A:B) < 1. In the *Pak2* ^−/−^ embryos, however, although the MHP was formed, the ratio of A: B at the DLHP remained > 2 (Figure 1E,F), suggesting the failure of DLHP formation. Moreover, the thickness of the neural tube in *Pak2* ^−/−^ embryos (65.360 ± 0.392 µm) was much smaller than that in WT embryos (121.6 ± 3.289 µm) (Figure 1G). Although *Pak2* ^+/−^ mice developed DLHPs (ratio of A: B, 0.640 ± 0.037), their neural tube thickness (102.6 ± 2.30 µm) was less than that of WT embryos, suggesting abnormal development. These results suggest that PAK2 is required for proper dorsolateral folding of the neural tube along the craniocaudal embryonic axis.

### 2.2 *Pak2* ^−/−^ Mouse Embryos Exhibit Upregulated BMP Signaling

**Figure 2:**
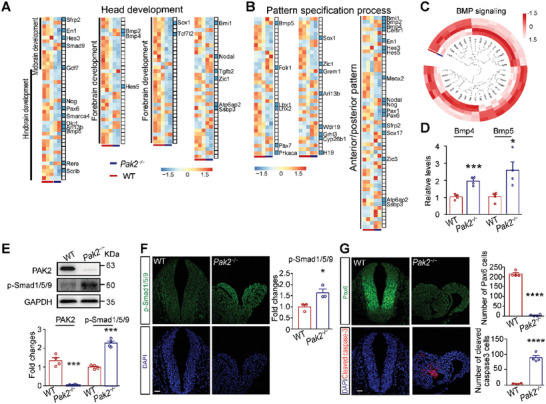
*Pak2* ^−/−^ mouse embryos exhibit upregulated BMP signaling. A,B) GO analysis indicates that DEGs are enriched in head development and pattern specification processes. Blue bars indicate NTD‐related genes. Scale bar: transcripts per million (TPM) after log2 transforms and Z‐score normalization. C) DEGs that are closely related to BMP signaling in *Pak2* ^−/−^ embryos. D) The levels of key components of BMP signaling, including *Bmp4* and *Bmp5*, in WT and *Pak2* ^−/−^ embryos at E9.5. *Bmp4*: t (6) = 6.112, *p* = 0.0009; *Bmp5*: t (6) = 3.06, *p* = 0.0222. *n* = 4 embryos for each genotype. E) The level of PAK2 and p‐Smad1/5/9 (Ser465) in *Pak2* ^−/−^ embryos as shown via western blotting (for PAK2, WT, 1.347 ± 0.151; *Pak2* ^−/−^ mice, 0.059 ± 0.009; *t* (6) = 8.542, *p* = 0.0001; for p‐Smad1/5/9, WT, 1 ± 0.057; *Pak2* ^−/−^ mice, 2.272 ± 0.109; t (6) = 10.35, *p* < 0.0001) of the hindbrain of embryos at E9.5. *n* = 4 embryos for each genotype. F) The level of p‐Smad1/5/9 (Ser465) in *Pak2* ^−/−^ mice as shown via immunofluorescence analysis of the hindbrain of embryos at E9.5. WT, 1 ± 0.106; *Pak2* ^−/−^ mice, 1.632 ± 0.170; *t* (4) = 3.161, *p* = 0.034. *n* = 3 embryos for each genotype. G) The numbers of Pax6‐positive cell (WT, 214.3 ± 6.921; *Pak2* ^−/−^ mice, 4.5 ± 2.021; *t* (6) = 29.09, *p* < 0.0001) and cells stained with cleaved caspase‐3 (WT, 3.250 ± 0.750; *Pak2* ^−/−^ mice, 88.25 ± 7.028; *t* (6) = 12.03, *p* < 0.0001) in the hindbrain of WT and *Pak2* ^−/−^ embryos at E9.5. *n* = 4 embryos for each genotype. Unpaired *t* test. Scale bar: 20 µm.

To investigate how PAK2 affected neural tube closure, RNA‐seq was performed on WT (3 embryos in one replicate, 3 replicates) and *Pak2* ^−/−^ (3 embryos in one replicate, 2 replicates) bulk embryos at E9.5. A total of 2888 differentially expressed genes (DEGs, *p* adjusted < 0.05, |log2 fold change| > 0.5), including 1402 upregulated and 1486 downregulated genes, were identified in *Pak2* ^−/−^ embryos (Table S1, Supporting Information). Gene ontology (GO) analysis of the DEGs in *Pak2* ^−/−^ bulk embryos identified head development, pattern specification process, spinal cord development, somite development, and synapse organization among the enriched terms (**Figure** **2**A,B and Figure S1A,B, Supporting Information). Among these DEGs, 91 were identified as NTD‐related genes (Table S2 and Figure S1C, Supporting Information). In particular, BMP signaling, including key components *Bmp4* and *Bmp5*, was upregulated in *Pak2* ^−/−^ embryos (Figure 2C,D). Moreover, the level of p‐Smad1/5/9 (Ser465), the main downstream effector of BMP signaling, was significantly increased in *Pak2* ^−/−^ embryos as determined by western blotting (Figure 2E) and immunofluorescence analysis of the hindbrain of embryos at E9.5 (Figure 2F). Taken together, these results suggest that BMP signaling is activated by *Pak2* deletion.

Considering that cell proliferation is required for neural tube closure and that BMP signaling inhibits cell proliferation,^[^ ^22^ ^]^ WT and *Pak2* ^−/−^ embryos were stained by immunofluorescence for Pax6, a regulator of cell proliferation, which also represented a downregulated DEG in *Pak2* ^−/−^ embryos (Table S1, Supporting Information). We found that the number of Pax6‐positive cells was dramatically decreased in *Pak2* ^−/−^ embryos compared with that in WT embryos (Figure 2G). In contrast, *Pak2* ^−/−^ embryos exhibited increased cell death in the neural tube as shown by cleaved caspase‐3 staining (Figure 2G), suggesting that enhanced BMP signaling inhibits cell proliferation and induces cell death in the neural tube.

### 2.3 Single‐Cell Transcriptomes Reveal Abnormal Differentiation Trajectories in *Pak2* ^−/−^ Embryos

**Figure 3:**
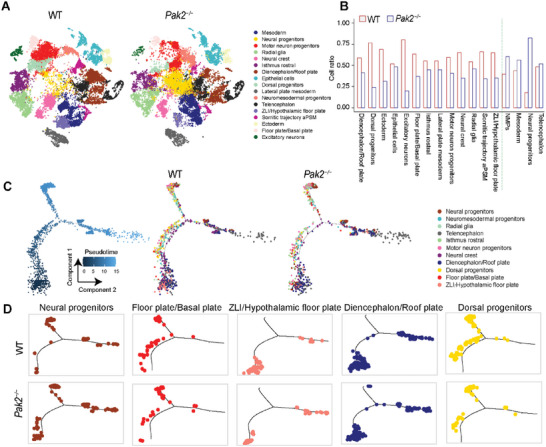
Abnormal developmental process in *Pak2^−/−^* embryos. A) The annotation and color codes for cell clusters in WT and *Pak2^−/−^* embryos at E9.5 as revealed by scRNA‐seq analysis. B) Cell ratio in different cell cluster between WT and *Pak2* ^−/−^ embryos. C) Pseudotime‐ordered analysis of 11 cell types related to neural tube in WT and *Pak2^−/−^* embryos. The blue trajectory includes cell types in both WT and *Pak2^−/−^* samples. D) Representative developmental trajectory of cell types from WT and *Pak2^−/−^* samples via pseudotime‐ordered analysis.

To define cell types and transcriptional events in the developing neural tube regulated by PAK2, we performed single‐cell RNA‐seq of WT and *Pak2* ^−/−^ embryos at E9.5 (3 embryos in one replicate, 2 replicates for each genotype) by using the DNBelab C4 platform.^[^ ^23^, ^24^ ^]^ After quality filtering, a dataset of 48060 cells (25581 cells for WT embryos and 22479 cells for *Pak2* ^−/−^ embryos, median unique molecular identifier (UMI) count, 3700; median genes detected, 1731) was retained for further analysis after applying quality filters. We first allocated cells to different tissues, different domains of progenitors, and neurons based on the combinatorial expression of molecular markers (Figure S2A,B, Supporting Information).^[^ ^25^, ^26^, ^27^, ^28^ ^]^ This allowed the classification of 45052 cells (23784 cells for WT embryos and 21268 cells for *Pak2* ^−/−^ embryos), 93.74% of the total cells, which were divided into 17 anticipated clusters through visualizing the resulting dataset with t‐distributed stochastic neighbor embedding (tSNE) (Figure S2C, Supporting Information). The scRNA‐seq analysis indicated that the *Pak2* level was dramatically downregulated in the majority of annotated cells and tissues in *Pak2* ^−/−^ embryos, suggesting its essential regulation of embryonic development (Figure S2D, Supporting Information). In WT embryos, epithelial cells, mesoderm cells, neural crest, neuromesodermal progenitors (NMPs), and motor neuron progenitors were abundant at E9.5, which was consistent with the initially high differentiation rate of embryos.^[^ ^29^, ^30^ ^]^ Compared with WT embryos, the cell ratios of most cell types in the neural tube and its developed forebrain, hindbrain, and spinal cord were decreased in *Pak2* ^−/−^ embryos (**Figure** **3**A,B), indicating their abnormal proliferation and differentiation.

In contrast, cell types related to early developmental stages, including mesoderm, NMPs, and neural progenitors, were increased in *Pak2* ^−/−^ embryos, indicating their retarded development (Figure 3A,B). The neural progenitors that are derived from the bipotent progenitor NMPs can contribute to the formation of preneural tube.^[^ ^31^, ^32^ ^]^ The increased number of neural progenitors in *Pak2* ^−/−^ embryos indicated the indispensable role of PAK2 in neural progenitor differentiation and preneural tube formation. In addition, considering that NMPs generate both spinal cord and paraxial mesoderm,^[^ ^31^, ^32^ ^]^ these results also suggested the essential role of PAK2 in spinal cord development.

To further evaluate PAK2‐regulated cell differentiation and developmental state of neural tube, the pseudotime analysis at a threshold of *q* value < 0.0001 was performed to order cell populations of WT and *Pak2^−/−^* embryos from the early to the late differentiation state along a developmental trajectory (Figure 3C).^[^ ^33^ ^]^ Consistently, the cell numbers in the neural progenitor trajectory showed a remarkably increase in most developmental stages in *Pak2^−/−^* embryos as compared to the cells in the WT embryos. On the contrary, *Pak2* deletion induced attenuated development in the floor plate/basal plate, zona limitans intrathalamica (ZLI)/hypothalamic floor plate, diencephalon/roof plate, and dorsal progenitor trajectory (Figure 3D). In addition, the development of radial glial that arise during the expansion of the neural tube at E9.5 and neural crest cells that originate at the dorsal edge of the neural tube were also dampened in *Pak2^−/−^* embryos (Figure S2E, Supporting Information).

### 2.4 Transcriptional Changes along with Abnormal Differentiation Trajectories in *Pak2* ^−/−^ Embryos

**Figure 4:**
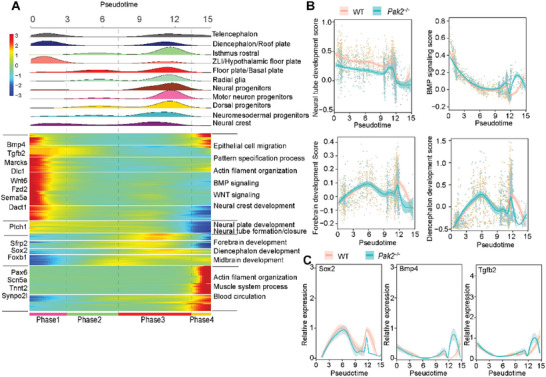
Abnormal transcriptional changes along with abnormal developmental process in *Pak2* ^−/−^ embryos. A) Heatmap showed the dynamic changes in gene expression along the pseudotime (lower panel). Cell types are labeled by colors (upper panel). B) 2D plots show the expression scores related to neural tube development, BMP signaling, forebrain development, and diencephalon development along the pseudotime. C) 2D plots show the dynamic expression of representative genes during neural tube development along the pseudotime.

To investigate the transcriptional changes associated with abnormal differentiation process, the above‐mentioned cells were categorized into 4 pseudotime phases to link neural cell fates to co‐regulated genes in specific developmental processes (**Figure** **4**A). Among them, genes in phase 1 cells were predominantly involved in the biological process of pattern specification, epithelial tube morphogenesis, and neural crest development; genes in phase 2 cells were mainly associated with neural tube developmental process, including primary neural tube formation and neural plate development and formation; genes in phase 3 cells were predominantly related to the function of forebrain, telencephalon, hypothalamus, and midbrain development. Genes in phase 4 were associated with the process of muscle system and blood circulation (Figure 4A and Table S3, Supporting Information). The dynamic expressions of genes during the biological process of neural tube development, BMP signaling, forebrain, and diencephalon development along the pseudotime were shown in Figure 4B. Compared to WT embryos, *Pak2^−/−^* embryos showed obviously lower scores of neural tube development along the pseudotime phase 1 and 2 (Figure 4B). Lower development scores of forebrain, particularly diencephalon, were mainly evident in pseudotime phase 3 in *Pak2^−/−^* embryos. The enriched molecular events included BMP and WNT signaling as well as actin filament assembly and organization (Figure 4A). In particular, the upregulated expression in the BMP signaling, such as *Bmp4* and *Tgfb2*, and downregulated expression of *Sox2* implied defected differentiation and development in *Pak2^−/−^* embryos. The dynamic expressions of *Sox2*, *Bmp4*, and *Tgfb2* along the pseudotime were shown in Figure 4C. These results together indicated abnormal transcriptional changes along with abnormal differentiation trajectories in *Pak2^−/−^* cells.

### 2.5 Identification of Two Nonsynonymous and One Recurrent Splice Site Mutation in the *PAK2* Gene in Human Fetuses with NTD

**Figure 5:**
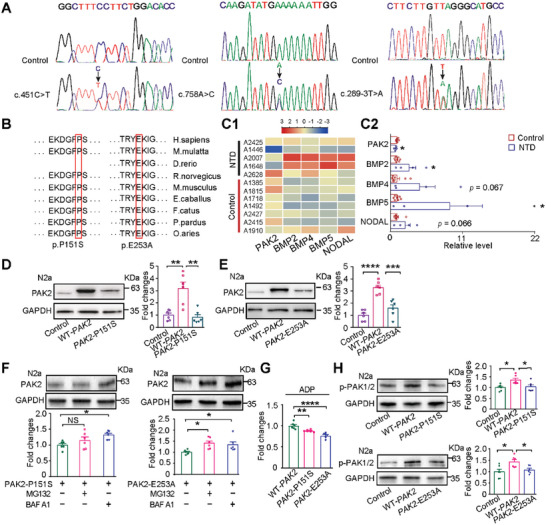
Identification of *PAK2* mutations in human fetuses with NTDs. A) Two nonsynonymous (c.451C>T, p.P151S; c.758A>C, p.E253A) and one recurrent splice site mutation (c.289‐3T>A) in the *PAK2* gene are identified among five patients with NTDs and validated using Sanger sequencing. B) Sequence conservation analysis of the amino acid sequences at P151 and E253 of PAK2 among multiple vertebrates. C1–C2) The relative levels of *PAK2* and key components in BMP signaling in brain tissues of health controls and human NTDs carrying *PAK2* mutations as shown using the NanoString nCounter RNA assay. C1, Color bar represents log2 fold‐change; C2, the relative levels of each gene are presented as the mean ± SEM. D) The levels of PAK2 (F = 7.612, *p* = 0.0052; *p* < 0.01 for WT‐*PAK2* versus *PAK2*‐P151S) in N2a cells transfected with WT‐*PAK2* and *PAK2*‐P151S. E) The levels of PAK2 (*F* (2, 15) = 26.41, *p* < 0.0001; *p* = 0.0002 for WT‐*PAK2* versus *PAK2*‐E253A) in N2a cells transfected with WT‐*PAK2* and *PAK2*‐E253A. F) The levels of PAK2 in N2a cells transfected with *PAK2*‐P151S (*p* = 0.298 for MG132; *p* = 0.016 for BAF A1) or *PAK2*‐E253A (*p* = 0.019 for MG132, *p* = 0.049 for BAF A1) were treated with MG132 or Baf‐A1 for 6 h. G) The levels of ADP in N2a cells transfected with WT‐*PAK2*, *PAK2*‐P151S and *PAK2*‐E253A. *p* = 0.007 for WT‐*PAK2* versus *PAK2*‐P151S, *p* < 0.0001 for WT‐*PAK2* versus *PAK2*‐E253A. H) The levels of pPAK2 (Ser141) in N2a cells transfected with WT‐*PAK2*, *PAK2*‐P151S and *PAK2*‐E253A. *p* = 0.026 for WT‐*PAK2* versus *PAK2*‐P151S, *p* = 0.036 for WT‐*PAK2* versus *PAK2*‐E253A. One‐way ANOVA with Dunnett's multiple comparison test (D–H). *n* = 6 cultures for each group.

To establish a direct relationship between *PAK2* and NTD pathology, we reanalyzed the genetic variation based on whole genome sequencing data in 100 Chinese fetuses with NTDs^[^ ^34^ ^]^ and identified a nonsynonymous mutation (c.451C>T, p.P151S) and one recurrent splice site mutation (c.289‐3T>A) in the *PAK2* gene in three independent patients. We further sequenced all *PAK2* coding regions in additional 216 human NTDs and identified a nonsynonymous mutation (c.758A>C, p.E253A) and the same splice site mutation (c.289‐3T>A) in the *PAK2* gene in two fetuses, resulting in a total of two nonsynonymous and one recurrent splicing variant detected in five individuals (**Table** **1**); these variants were subsequently validated by Sanger sequencing (**Figure** **5**A). Notably, these mutations represented extremely rare variants and were not found in the dbSNP147, ESP6500, 1000 Genome, or ExAC databases. Sequence conservation analysis showed that the amino acid sequences at P151 and E253 of PAK2 protein were conserved across multiple vertebrates (Figure 5B and Figure S3A, Supporting Information). The nucleotide sequence at T289‐3T was also conserved across multiple vertebrates (Figure S3B, Supporting Information). In addition, the online program “splice prediction by neuronal network”^[^ ^35^, ^36^ ^]^ predicted that the splice site mutation (c.289‐3T>A) might abolish the efficiency of the natural splice site of *PAK2*, with a score shift from 0.92 to 0.

**Table 1:** Clinical characteristics of *PAK2* rare variants in human NTD cases

Patient ID	Gestational age	Gender	NTD phenotypes	Variants
A2425	26W	F	Thoracic lumbar sacral spina bifida aperta (4.1 cm × 0.6–2.9 cm)	NM_002577:exon8:c.A758C:p.E253A
A2007	20W	M	Anencephaly/occipital cervical spina bifida aperta (2.7 cm × 2.4 cm)	NM_002577:exon5:c.451C>T:p.P151S
A1648	22W	M	Anencephaly/huge occipital encephalocele (6.5 cm × 4.5 cm × 0.8 cm)	NM_002577:exon4:c.289‐3T>A
A1446	20W	M	Lumbar sacral spina bifida aperta (3.5 cm × 2.0 cm)	NM_002577:exon4:c.289‐3T>A
A1594	31W	F	Occipital cervical thoracic spina bifida aperta (4 cm × 1.3 cm)	NM_002577:exon4:c.289‐3T>A

In the comprehensive population genome variation database (Chinese Millionome Database, CMDB), which included the whole‐genome sequencing data of 141431 unrelated healthy Chinese individuals, only one missense mutant in the *PAK2* gene was identified.^[^ ^37^ ^]^ The two missense mutants *PAK2*‐P151S and *PAK2*‐E253A were not found in CMDB.^[^ ^37^ ^]^ In addition, the *observed/expected (oe)* constraint score of *PAK2* gene according to the version of gnomAD (v2.1) was 0.14, which was lower than the upper bound of the *oe* confidence interval *<* 0.35. The data together suggested that *PAK2* was highly intolerant to functional genetic variants.

To explore whether the identified *PAK2* mutations affected the expression of *PAK2* and the core components of BMP signaling, four NTD fetuses with *PAK2* mutations (two carrying missense mutations and two carrying splice site mutations) and their gestational age‐matched controls (1:2) were evaluated using the NanoString nCounter RNA assay. The results showed that expression of the *PAK2* gene was downregulated in the brain of NTDs carrying *PAK2* mutations compared with that in control tissues (Figure 5C). Similar to *Pak2* ^−/−^ mouse embryos, we observed the overall activated BMP signaling including upregulated *BMP2*, *BMP4*, *BMP5*, and *NODAL* in the human NTDs carrying *PAK2* mutations (Figure 5C1,C2). Together, these findings suggest that *PAK2* deficiency was associated with upregulated BMP signaling in human NTDs.

To investigate the effects of the identified mutations on PAK2 levels, we overexpressed *PAK2*‐P151S and *PAK2*‐E253A in Neuro‐2A (N2a) cells. Compared to those in cells overexpressing the WT‐*PAK2* plasmid, the levels of PAK2 were dramatically decreased in *PAK2*‐P151S (Figure 5D) and *PAK2*‐E253A transfected cells (Figure 5E). These results suggest that *PAK2*‐P151S and *PAK2*‐E253A mutations attenuated PAK2 expression. To investigate whether the proteasome system or autophagy contribute to the instability of PAK2 protein, N2a cells overexpressed with *PAK2*‐P151S or *PAK2*‐E253A plasmids were treated with a proteasome inhibitor (MG132) or an autophagy inhibitor (bafilomycin A1, Baf‐A1) for 6 h. As a result, both MG132 and Baf‐A1 treatment markedly increased PAK2 accumulation in N2a cells transfected with the *PAK2*‐E253A; whereas only MG132 treatment increased the PAK2 level in N2a cells transfected with *PAK2*‐P151S (Figure 5F). These results suggest that the protein degradation pathways contribute to the instability of PAK2‐P151S and PAK2‐E253A proteins.

To examine the effects of *PAK2* mutations on the kinase activity, an ADP ELISA kit was employed to detect the concentration of ADP due to that PAK2 are enzymes using ATP as the source of phosphate. Compared with cells overexpressed with WT‐*PAK2*, the ADP levels were significantly decreased in cells transfected with *PAK2*‐P151S or *PAK2*‐E253A (Figure 5G). Consistent with this, the levels of p‐PAK2 (Ser141), an active form of the protein,^[^ ^38^ ^]^ were also significantly decreased in cells transfected with *PAK2* mutants (Figure 5H). These results together suggest that *PAK*2‐E253A and *PAK2*‐P151S mutations attenuated kinase activity.

### 2.6 PAK2 Regulates Smad9 Phosphoration to Inhibit BMP Signaling

**Figure 6:**
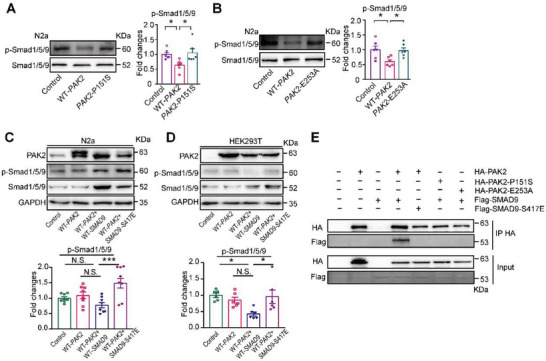
PAK2 regulates Smad9 phosphorylation to inhibit BMP signaling. A) The levels of p‐Smad1/5/9 (Ser 465) (*F* (2, 15) = 5.185, *p* = 0.019; for WT‐*PAK2* versus *PAK2*‐P151S, *p* = 0.0185) in N2a cells transfected with WT‐*PAK2* and *PAK2*‐P151S. *n* = 6 cultures for each group. B) The levels of p‐Smad1/5/9 (Ser 465) (F (2, 15) = 5.33, *p* = 0.018; for WT‐*PAK2* versus *PAK2*‐E253A, *p* = 0.029) in N2a cells transfected with WT‐*PAK2* and *PAK2*‐E253A. *n* = 6 cultures for each group. C) The levels of p‐Smad1/5/9 (Ser465) in N2a cells overexpressing WT‐*PAK2*, WT‐*PAK2*/WT‐*SMAD9*, and WT‐*PAK2*/*SMAD9*‐S417E plasmids. *F* (3, 28) = 7.393, *p* = 0.0009; for WT‐*PAK2*/WT‐*SMAD9* versus WT‐*PAK2*/*SMAD9*‐S417E, *p* = 0.0004, *n* = 8 cultures for each group. D) The levels of p‐Smad1/5/9 (Ser465) in HEK293T cells overexpressing WT‐*PAK2*, WT‐*PAK2*/WT‐*SMAD9*, and WT‐*PAK2*/*SMAD9*‐S417E plasmids. *F* (3, 20) = 4.833, *p* = 0.011, WT‐*PAK2*/WT‐*SMAD9* versus WT‐*PAK2*/*SMAD9*‐S417E, *p* = 0.025. *n* = 6 cultures for each group. E) Coimmunoprecipitation of Smad9 with PAK2, whereas *SMAD9*‐S417E, *PAK2*‐P151S, and *PAK2*‐E253A fail to interact with PAK2 and Smad9, respectively. One‐way ANOVA with Dunnett's multiple comparison test (A,B) and with Tukey's multiple comparison test (C,D).

Notably, we found that the levels of p‐Smad1/5/9 (Ser 465) were dramatically increased in both *PAK2*‐P151S and *PAK2*‐E253A transfected cells compared with those in WT‐*PAK2*‐transfected cells (**Figure** **6**A,B). Conversely, the levels of p‐Samd1/5 protein did not significantly change in cells transfected with the *PAK2*‐P151S or *PAK2*‐E253A plasmids (Figure S4A,B, Supporting Information), suggesting that PAK2 may regulate Smad9 phosphorylation to inhibit BMP signaling. As a serine/threonine protein kinase, PAK2 phosphorylates its targets on KRX[S/T] motif,^[^ ^39^ ^]^ which exits upstream of Ser417 of Smad9. In addition, it has been reported that Smad2 phosphorylation at Ser417 affects the level of p‐Smad1/5/9 (Ser465) by altering the L3 loop conformation of Smad9.^[^ ^40^ ^]^ Considering that Smad9 shares sequence similarity with Smad2, especially at the sites of Ser417 and Ser465, we wondered whether PAK2 regulated Smad9 phosphorylation to inhibit BMP signaling. By replacing the amino acid serine of Smad9 at position 417 with glutamic acid (E), the levels of p‐Smad1/5/9 (Ser465) were increased in cells transfected with both WT‐*PAK2* and *SMAD9*‐S417E plasmids, compared to those in N2a cells co‐transfected with WT‐*PAK2* and WT‐*SMAD9* plasmids (Figure 6C). Consistent with this, the level of p‐Smad1/5/9 (Ser465) was also increased in HEK293T cells overexpressing the WT‐*PAK2* and *SMAD9*‐S417E plasmids (Figure 6D). Conversely, we found that the levels of p‐Smad1/5 did not differ between cells co‐transfected with WT‐*PAK2*/*SMAD9‐*S417E and WT‐*PAK2*/WT*‐SMAD9* plasmids for either N2a or HEK293T cells (Figure S4C,D, Supporting Information).

To explore whether PAK2 directly interacts with p‐Smad9, CoIP experiments were carried out using whole cell lysates, which were immunoprecipitated with an anti‐HA‐PAK2 antibody and immunoblotted with the corresponding antibodies. We found that SMAD9 coimmunoprecipitated with PAK2, whereas *SMAD9*‐S417E did not (Figure 6E), suggesting that PAK2 interacts with Smad9 at Ser417. Moreover, *PAK2*‐P151S and *PAK2*‐E253A also failed to interact with Smad9 (Figure 6E).

### 2.7 Depletion of *pak2a* Resulted in Malformation of Neural Tube in Zebrafish

**Figure 7:**
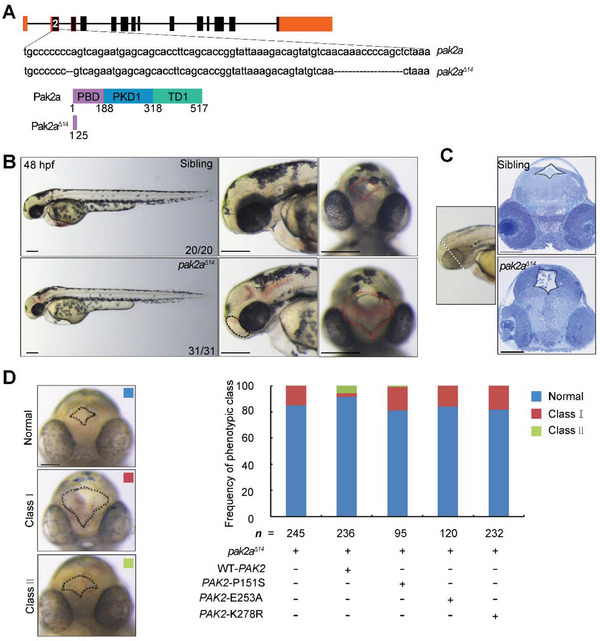
Depletion of *pak2a* in zebrafish induces neural tube defects, which were partially rescued by overexpression of WT‐*PAK2*, but not *PAK2* mutations. A) The *pak2a* mutant *pak2a* ^Δ14^, which contains a 14 bp deletion in the second exon, is generated using the CRISPR‐Cas9 system. B,C) An overt cavity in the third ventricle and diencephalon and a hemorrhage phenotype are observed in *pak2a* ^Δ14^ mutants at 48 hpf. *n* = 20 for sibling, *n* = 31 for *pak2a* ^Δ14^ mutants. D) The proportion of embryos with neural tube defects injected with mRNAs harboring human WT‐*PAK2*, *PAK2*‐P151S, *PAK2*‐E253A, and *PAK2*‐K278R mutants. *n* = 245 embryos for *pak2a* ^Δ14^ without injection, *n* = 236 embryos for *pak2a* ^Δ14^ with WT‐*PAK2* injection, *n* = 95 embryos for *pak2a* ^Δ14^ with *PAK2*‐P151S injection, *n* = 120 embryos for *pak2a* ^Δ14^ with *PAK2*‐E253A injection, *n* = 232 embryos for *pak2a* ^Δ14^ with *PAK2*‐K278R injection. Scale bar: 200 µm (B,C), 100 µm (D).

A previous study has indicated that Pak2a in zebrafish, which is broadly expressed in the brain, spinal cord, and endoderm of zebrafish, shares the highest homology in amino acid sequence with human PAK2, therefore considered as an ortholog of human PAK2.^[^ ^41^ ^]^ To genetically define the function of *pak2* in neural tube formation, we generated *pak2a* zebrafish mutants using the CRISPR‐Cas9 system. The generated mutant, named *pak2a* ^Δ14^, contained a 14 bp deletion in the second exon that led to the truncation of Pak2a, resulting in a protein reflecting only the first 25 amino acids of Pak2a (**Figure** **7**A). The *pak2a* ^Δ14^ homozygous embryos were indistinguishable from WT siblings up to 48 hours post‐fertilization (hpf), after which they showed hemorrhage in the diencephalon, midbrain, and hindbrain (Figure 7B), a phenotype consistent with the previous report.^[^ ^41^ ^]^

We further evaluated the neural tube morphology of *pak2a* ^Δ14^ embryos at 48 hpf and identified a cavity in the anterior‐dorsal cranial region (Figure 7B), suggestive of defective neural tube closure. Histological sectioning further revealed an overt cavity in the third ventricle and diencephalon in *pak2a* ^Δ14^ mutant embryos at 48 hpf (Figure 7C). Moreover, red blood cells were concurrently apparent in the third ventricles (Figure 7C).

Next, we employed the *pak2a* ^Δ14^ mutants to evaluate the functional consequences of the *PAK2* mutations identified in human NTDs. In *pak2a* ^Δ14^ F1 generation incrosses, the proportion of embryos with defects was approximately 18% (Figure 7D). However, when in vitro‐synthesized mRNA encoding human WT‐PAK2 was injected into *pak2a* ^Δ14^ embryos at the 1‐cell stage, only 4% of the resulting embryos were phenotypically *pak2a* ^Δ14^ mutant, whereas 5% were partially rescued, demonstrating the functional conservation of the human and zebrafish *PAK2* genes (Figure 7D). In contrast, injection of mRNAs containing the human *PAK2*‐P151S and *PAK2*‐E253A missense mutations resulted in 18% and 16% *pak2a* ^Δ14^ mutants, respectively, implying that these two mutations disrupted the ability to rescue the defects and were loss‐of‐function mutations (Figure 7D). Previous studies have indicated that the *PAK2*‐K278R mutant is deficient in kinase activity of PAK2 and thus referred to as the kinase‐dead mutant.^[^ ^42^, ^43^ ^]^ To examine the rescue effects of *PAK2* kinase‐dead mutant, *pak2a* ^Δ14^ zebrafish embryos were injected with *PAK2*‐K278R mRNAs. We found that 18% of *pak2a* ^Δ14^ zebrafish embryos injected with *PAK2*‐K278R mRNAs showed abnormal neural tube closure, which was similar to the effects of *PAK2*‐P151S and *PAK2*‐E253A, indicating that *PAK2* kinase‐dead mutant failed to rescue the neural tube defects of *pak2a* ^Δ14^ zebrafish embryos. Taken together, these results showed that Pak2a participated in the formation of neural tubes in zebrafish.

## 3 Discussion

As a member of the group I PAKs, PAK2 has been found to regulate endothelial development and neuronal functions.^[^ ^8^, ^10^ ^]^ However, its role in neuroepithelial development and its related downstream signaling have not yet been investigated in detail. In this study, we found that *Pak2* ^−/−^ mouse embryos failed to develop DLHPs and exhibited an open neural tube. *Pak2* homozygous deletion induced BMP signaling, including upregulation of its key components and the main downstream effector Smad9. *Pak2* knockout also induced abnormal developmental trajectory and transcriptional events in the developing neural tube. Moreover, human NTDs that carried deleterious *PAK2* mutations exhibited attenuated *PAK2* expression and activated BMP signaling in the brain. We further showed that PAK2 regulated Smad9 phosphorylation to inhibit BMP signaling. Together, our results revealed that the PAK2‐mediated BMP signaling is essential for neural tube closure in multiple model systems.

BMPs constitute members of the transforming growth factor (TGF)‐*β* family and exert diverse functions during embryogenesis and nervous system development.^[^ ^11^ ^]^ In particular, loss of Noggin, a BMP antagonist, resulted in somite development deficits and failures to develop DLHPs in the upper spinal cord.^[^ ^19^, ^44^, ^45^ ^]^ The lack of DLHPs further led to a failure of neurulation in the midbrain and hindbrain and thus the exencephaly phenotype.^[^ ^45^ ^]^ Increased BMP signaling, particularly BMP4, was found to contribute to the above phenotypes.^[^ ^45^ ^]^ In the present study, we found the similar phenotypes in *Pak2* ^−/−^ mouse embryos, which showed somite development deficits and craniorachischisis due to the lacking of DLHPs. Bulk RNA‐seq, single‐cell RNA‐seq, and NanoString nCounter RNA assay consistently showed that increased *Bmp4* levels were associated with PAK2 dysfunction in both mouse embryos and human NTDs. BMP4 is specifically expressed in the diencephalic roof plate;^[^ ^46^ ^]^ consistent with this, abnormal diencephalon development was also found in *pak2a* ^Δ14^ zebrafish embryos. Other components in BMP signaling, such as upregulated *Bmp2* and *Nodal*, were also manifested in human NTD fetuses carrying *PAK2* mutations.

Single‐cell RNA‐seq analysis further indicated upregulated BMP signaling associating with abnormal developmental trajectory in *Pak2* ^−/−^ mouse, suggesting that BMP signaling may exert function in the differentiation of specific cell types. Previous studies have found that loss of BMP signaling reduced mesodermal cell identity.^[^ ^47^ ^]^ Moreover, mesoderm area expansion can drive MHP formation, whereas DLHPs develop in regions with low mesoderm expansion.^[^ ^48^ ^]^ Considering that MHP but not DLHP develops in *Pak2* ^−/−^ embryos, it is possible that BMP signaling blocks DLHP formation through shifting bipotent NMPs toward mesoderm, as increased mesoderm but decreased epithelial cells in *Pak2^−/−^* mouse embryos.

Neural tube closure requires adequate proliferation in the neuroepithelium owing to the rapid growth of the embryo during neurulation. Conversely, single‐cell RNA‐seq analysis showed decreased numbers in multiple cell types in the spinal cord and forebrain trajectory, including motor neuron progenitors, dorsal progenitors, diencephalon/roof plate, and ZLI/hypothalamic floor plate in *Pak2* ^−/−^ embryos, suggesting reduced proliferation and differentiation in them. Consistent with this, abolished level of the cell differentiation marker Pax6 and increased cell death were evident in *Pak2* ^−/−^ mouse embryos. This may be also attributed to the activation of BMP signaling, which inhibited the expression of Pax6 and Pax7 (notably identified as downregulated DEGs), thereby preventing the establishment of dorsal identity in the neural tube.^[^ ^17^ ^]^ Together, these findings suggest that the enhanced BMP signaling contributes to neural tube deficits in *Pak2* ^−/−^ mouse embryos and human NTDs carrying *PAK2* mutations.

We noticed that actin cytoskeleton organization, a process regulated by PAK2, was also disturbed in pseudotime phase 1, suggesting that there were other factors contributing to the severe phenotype in *Pak2* ^−/−^ mouse embryos. In fact, a total of 91 DEGs in *Pak2* ^−/−^ mouse embryos were previously identified NTD‐related genes, representing 18.88% of the total NTD genes. Among them, the levels of genes encoding proteins associated with cell differentiation of the neural tube (e.g., Notch pathway genes *Hes3* and *Hes5*) were also altered in *Pak2* ^−/−^ mouse embryos. Moreover, abnormal WNT signaling was also evident in bulk RNA‐seq data of *Pak2* ^−/−^ mouse embryos and in human NTDs carrying *PAK2* mutations using the NanoString nCounter RNA assay (Figure S5, Supporting Information). Considering that the role of WNT/PCP signaling in the regulation of anterior/posterior patterning and neural tube development is well established,^[^ ^49^, ^50^ ^]^ how PAK2 regulates the WNT/PCP signaling in the neural tube also requires further study in the future.

Although hundreds of NTD‐associated genes have been reported in animal studies, there is very little genetic evidence of these genes linking to NTD in humans, leaving a gap between animal models and human genetics of NTD.^[^ ^2^, ^51^, ^52^ ^]^ However, *Pak2* ^−/−^ mouse embryos, human fetuses carrying *PAK2* mutations, and *pak2a* ^Δ14^ zebrafish embryos shared similarities in phenotypes. Thus, our study identified a conserved gene regulating neural tube development in multiple vertebrate species, which also provided a validated animal model for potential treatment study of NTD.

Previous studies have indicated that PAK2 dysfunctions are involved in the pathology of autism,^[^ ^8^ ^]^ 3q29 deletion syndrome,^[^ ^53^, ^54^ ^]^ cancer progression,^[^ ^7^ ^]^ and even flawed blood vessel formation.^[^ ^10^ ^]^ Consistent with this, blood circulation was among the top enriched function in genes in pseudotime phase 4, underlying the molecule mechanism of hemorrhage in *pak2a* ^Δ14^ zebrafish embryos.^[^ ^41^ ^]^ Moreover, heart development, artery development, and heart morphogenesis were also the enriched terms in genes in pseudotime phase 4. Considering that congenital heart defect is the most common type of birth defect, the role of PAK2 in embryonic heart development and its related abortion also requires further investigation.

In summary, by integrating multiple levels of evidence from human genetics, multiple model systems, and single‐cell transcriptomics, we demonstrate that the PAK2 regulates essential pathways for neural tube formation, highlighting the molecular pathogenesis of *PAK2* gene mutations in neural tube defects.

## 4 Experimental Section

### Human NTD Sample Collection

Stillborn NTD subjects were obtained from the Shanxi Province of northern China from 2004. The NTDs were classified using the International Classification of Diseases (ICD‐10). The enrolled pregnant women were diagnosed by trained local clinicians using ultrasonography. The surgical procedures were performed as previously described.^[^ ^55^ ^]^ The epidemiological method was described in detail in the previous publication.^[^ ^56^ ^]^ A total of 316 NTD subjects were included in this study. The control subjects, who had been aborted for nonmedical reasons, were also obtained from local hospitals. Any fetuses displaying pathologic malformation or intrauterine growth retardation were excluded from the control group. The investigation was approved by the Committee of Medical Ethics of the Capital Institute of Pediatrics. Written informed consent was obtained from all mothers who participated in this study (SHERLLM2019017).

### PAK2 Sequencing

Whole genome sequencing was performed on 100 NTD cases as previously described.^[^ ^34^ ^]^ Briefly, sequencing was performed on an Illumina 10 × platform (Illumina, San Diego, CA, USA) and the fastq files were mapped to the hg38 reference sequence. The average depth of the coverage was 30.2 ×. The remaining 216 NTD cases were sequenced using the target‐capture sequencing. Specifically, genomic DNA (200 ng) extracted from muscle (195 cases), lung (18 cases), or skin tissue (3 cases) of each individual was sheared using a Biorupter (Diagenode, Belgium) to acquire 150–200 bp fragments. The ends of the DNA fragment were repaired, and an Illumina Adaptor was added (Fast Library Prep Kit, iGeneTech, Beijing, China). Following construction of the sequencing library, whole genes were captured using the TargetSeq Enrichment Kit V1 (iGeneTech, Beijing, China) and sequenced on an Illumina platform with 150 base paired‐end reads. Raw reads were filtered using FastQC to remove low‐quality reads. Clean reads were then mapped to the reference genome GRCh37 using Bwa. After removing duplications, single nucleotide variants (SNVs) and insertions/deletions (InDels) were called and annotated using GATK. To validate the genotyping results of *PAK2* obtained from high‐throughput sequencing, the regions containing identified *PAK2* mutations in NTD samples were amplified by PCR and validated using Sanger sequencing.

### NanoString nCounter Assay

The NanoString nCounter assay (NanoString Technologies, Seattle, WA, USA) was used to detect the number of transcripts in human brain tissues, including those from four with NTD and potential detrimental *PAK2* mutants as well as from eight gestationally matched control individuals (1:2). Due to lack of age‐matched controls for the fetus (A1594) carrying the splice site mutation with the gestational age of 31weeks, it was not introduced to the NanoString nCounter RNA assay. Total RNA was extracted following the manufacturer's instructions (miRNeasy Mini Kit, Qiagen, Hilden, Germany) and processed using gene‐specific probes designed by the manufacturer (NanoString Technologies). Hybridization was performed according to the nCounter Element 24‐plex Assay Manual. An aliquot of each RNA sample (approximately 100 ng) was mixed with nCounter Reporter probes (20 µL) in hybridization buffer and nCounter Capture probes (5 µL) for a total reaction volume of 30 µL. The hybridizations were incubated at 65 °C for approximately 16 h, then eluted, and immobilized in the cartridge for data collection, which was performed on the nCounter Digital Analyzer. Gene expression data were filtered using quality control (QC) criteria according to the manufacturer's recommendations. Raw counts of QC‐passed samples were normalized using three reference genes as internal controls (*GAPDH*, *CLTC*, and *GUSB*). All QC and normalization procedures were performed using nSolver Analysis Software v2.0, and all data were log2‐transformed prior to further analysis.

### Immunostaining of Mouse Embryos

All procedures followed the guidelines of the National Institutes of Health and were approved by the Animal Usage Committee at the Institute of Zoology, Chinese Academy of Sciences. The *Pak2* gene was knocked out via gene trapping as previously described.^[^ ^8^ ^]^ *Pak2^−/−^* embryos and their littermate controls were dissected at E8.5‐10.5 in cold PBS‐bovine serum albumin (BSA) and fixed in paraformaldehyde (4%) at 4 °C overnight. Whole embryos were stained in DAPI Staining Solution (Cat^#^ C1006, Beyotime, Shanghai, China) for 20 min at room temperature.

Immunofluorescence analysis was performed according to standard protocols. For immunostaining of section, embryos were collected from pregnant mice at E9.5, post‐fixed in paraformaldehyde (4%) for 2 days, and processed for immunofluorescence as paraffin sections (5 µm). Briefly, embryonic sections were washed with PBS containing Triton X‐100 (0.1%) and blocked with PBS containing BSA (3%) for 60 min at room temperature. Sections were labeled overnight at 4 °C with primary antibodies diluted in PBS containing BSA (1%), washed with PBS, and then labeled for 3 h at 4 °C with a fluorescently labeled secondary antibody mixture in the same buffer. The primary antibodies included anti‐PAK2 (1:200; Cat^#^ 2608, Cell Signaling Technology, Danvers, MA, USA), anti‐p‐Smad1/5/9(D5B10) (1:800; Cat^#^ 13820, Cell Signaling Technology), anti‐Smad1/5/9 (1:400; Cat^#^ ab66737, Abcam, Abcam Trading (Shanghai) Company, Shanghai), anti‐cleaved‐caspase3 (1:200; Cat^#^ 9661, Cell Signaling Technology), and anti‐Pax6 (1:200; Cat^#^ ab5790, Abcam). In addition to the whole embryo immunofluorescence that was imaged using a Zeiss LSM 780 (Zeiss, Oberkochen, Germany), images of embryonic sections were acquired using an FV1000 microscope (Olympus, Tokyo, Japan) under the same conditions. The thickness of neural tube was obtained by measuring the distance from the apical to the basal membrane of the lateral neural plate. ImageJ was used to draw regions of interest (ROIs) around the hindbrain in the confocal images of stained embryo sections.

### RNA‐Seq and Bioinformatics Processing of the Data

Total RNA was extracted from whole embryos of WT and *Pak2* ^−/−^ mice at E9.5, using the RNeasy Mini Kit (Qiagen). Specifically, we performed two or three biological repeats for each genotype (WT and *Pak2* ^−/−^ mice) at E9.5. RNA quality was assessed using the Agilent Bioanalyzer 2100 RNA 6000 Nano Kit (Agilent Technologies, Santa Clara, CA, USA). The libraries were generated from total RNA via polyA^+^ selection of mRNA using the TruSeq RNA Sample Prep Kit v2 (Illumina), and transcriptomes were sequenced using the HiSeq 2000 Sequencing System (Illumina) in paired‐end mode.

Sequencing adapters and low‐quality sequencing reads were excluded using Trim Galore (http://www.bioinformatics.babraham.ac.uk/projects/trim_galore/). After that, clean PE reads were aligned against the mm10 transcriptome using HISAT2 (version 2.1.0) and quantitated with featureCounts (version 1.5.0‐p2). The software package edgeR (version 3.28.1) was employed for differential expression analysis with raw count. Genes with transcripts per million (TPM) > 0.25 in at least two samples were used for subsequent differential expression analysis. The cutoff of *p* adjusted < 0.05, |log2 fold change| > 0.5 were used for DEG identification. Enriched GO terms and KEGG pathway were identified using Metascape. Heatmaps of gene expressions (TPM), GO and KEGG pathway enrichment analyses for modules were carried out using the clusterProfiler (version 3.14.3) R package, iTOL (https://itol.embl.de/), or TBtools. Go terms and pathways with *p* value < 0.05 and minimum number of genes > 2 were defined as significantly enriched.

### Quantitative Real‐Time Polymerase Chain Reaction (RT‐PCR) Analysis

Total RNA was extracted from whole embryos of pregnant mice at E9.5, using the TRIzol reagent (Invitrogen). RT‐PCR was conducted using the Maxima SYBR Green qPCR Master Mix kit (CWBIO, Beijing, China) according to the manufacturer's instructions in an ABI Prism 7500 Sequence Detection System (Applied Biosystems, Thermo Fisher Scientific, Rockford, IL, USA) with the following primers: *Pak2*, forward primer, 5′‐CTTGGGTGGAGAGGCTATTC‐3′; reverse primer, 5′‐AGGTGAGATGACAGGAGATC‐3′. The relative mRNA levels of *Pak2* were normalized to those of *Gapdh* (forward primer, 5′‐CAAGCTCATTT CCTGGTATGAC‐3′; reverse primer 5′‐ CTGGGATGGAAATTGTGAGG‐3′).

### PAK2 Expression Constructs and Transient Transfection

The HA‐tagged WT‐*PAK2* construct has been described previously.^[^ ^8^ ^]^ Flag‐tagged WT Smad9 and kinase‐deficient (S417E) Smad9 were subcloned into the pcDNA3.0 (+) expression vector (Addgene, Cambridge, MA, USA). WT‐*PAK2*, *PAK2*‐P151S, *PAK2*‐E253A, and *PAK2*‐K278R containing *β*‐globin were then subcloned into the pcDNA3.0 (+) expression vector through standard restriction enzyme digestion and ligation and verified using Sanger sequencing. N2a or HEK293T cells were maintained in Dulbecco's modified Eagle's medium (GIBCO, Thermo Fisher Scientific) supplemented with fetal bovine serum (10%) (Invitrogen, Thermo Fisher Scientific) and penicillin/streptomycin (1%). The plasmids (2 µg) were transfected into cells using Lipofectamine 3000 Reagent (Thermo Fisher Scientific). Cells were harvested 48 h after transfection for protein extraction. The N2a cells transfected with *PAK2*‐P151S and *PAK2*‐E253A were treated with 2 µ MG132 (Cat^#^ HY‐13259, MedChemexpress) or 20 nm bafilomycin A1(BAF A1, Cat^#^ HY‐100558, MedChemexpress) for 6 h.

### Western Blots

For total protein extraction, the tissue and cell samples were lysed in Radio Immunoprecipitation assay (RIPA) lysis solution (CWBIO) with protease and phosphatase inhibitors (Roche, Madison, WI, USA), followed by denaturation at 95 °C for 10 min. The denatured supernatant was separated using sodium dodecyl sulfate‐polyacrylamide gel electrophoresis (12%) and transferred to polyvinylidene difluoride membranes at 4 °C for 90 min. The membranes were blocked with BSA (5%) or defatted milk (5%) and incubated with primary antibody in BSA (5%) at 4 °C overnight. The primary antibodies were obtained from Cell Signaling Technology, including anti‐PAK2 (1:1000; Cat^#^ 2608), anti‐p‐Smad1/5/9 (D5B10) (1:1000; Cat^#^ 13820), anti‐p‐Smad1/5(41D10) (1:1000; Cat^#^ 9516), and anti‐p‐PAK1 (Ser144)/PAK2 (Ser141) (1:1000; Cat^#^ 2606); and from Abcam, including Smad1/5/9 (1:1000; Cat^#^ ab66737). The secondary antibodies used were anti‐rabbit IgG conjugated to horse radish peroxidase (HRP) (Cat^#^ 7074, Cell Signaling Technology) and anti‐mouse IgG conjugated to HRP (Cat^#^ 7076, Cell Signaling Technology). Signals were detected using the FluorChem E imaging system (Cell Biosciences, San Jose, CA, USA) and analyzed using ImageJ software (version 1.4.3.67). The relative levels of these proteins were normalized to that of GAPDH (1:5000; Cat^#^ 60004, Proteintech, Rosemont, IL, USA).

### Adenosine Diphosphate Concentration Detection

According to the instructions of the ADP ELISA kit (Shanghai Enzyme‐linked Biotechnology, China), the lysed supernatant was collected from N2a cells transfected with WT‐*PAK2*, *PAK2*‐P151S, and *PAK2*‐E253A plasmids for 48 h. The ADP level from 1 µg of the samples was measured by the optical density (OD) value at 450 nm. The concentration of ADP in the samples was then determined by comparing the OD of the samples to the standard curve.

### scRNA‐seq

WT and *Pak2^−/−^* embryos (*n* = 2 replicates for each genotype) were collected on E9.5. Each embryo was washed twice with pre‐cooled D‐PBS, then cut into small pieces (0.2–0.5 mm^3^) with scissors in D‐PBS (1 mL) containing fetal bovine serum (1%) (GIBCO). The small pieces were digested into single‐cell suspensions with PAPAIN (40 µL, 10 mg mL^−1^; Cat^#^ 10108014001, Sigma, St. Louis, MO, USA) and DnaseI (5 µL, 10 U µL^−1^; Cat^#^ CD4871, Beijing Cool Laibo Technology, Beijing, China) at 37 °C for 30 min. After dissociation, the cell suspensions were filtered using a cell strainer (40 µm) (BD Falcon, Thomas Scientific, Swedesboro, NJ, USA), and PBS was added. Centrifugation was performed at 300 g for 5 min. After the supernatant was discarded, cell activity and number were calculated upon resuspension in D‐PBS (1 mL). Single‐cell 3′ mRNA transcriptome profiling was performed using a negative‐pressure orchestrated DNBelab C4 system (MGI Tech, Shenzhen, China) according to the workflow.

For all samples, PISA was used to perform sample de‐multiplexing, barcode processing, and single‐cell 3′ unique molecular identifier (UMI) counting with default parameters. Processed reads were then aligned to the complete mouse reference genome (mm10) using STAR splicing‐aware aligner with default parameters. Valid cells were automatically identified based on the UMI number distribution in each cell.

To obtain high‐quality cells, we set the following criteria: number of genes in each cell in the range of 500 to 6000, ratio of mitochondrial genes < 0.05, and number of UMIs > 2000. To avoid unexpected noise, genes detected in fewer than three cells were excluded. A total of 45052 high‐quality cells were selected for further analysis. DoubletFinder was used to remove the doublets from each sample in the dataset. The gene expression matrices were normalized by applying the “Regress Out” function in Seurat to the total cellular read count and mitochondrial read count using linear regression. Highly variable genes identified with the “FindVariableGenes” function were used to perform principal component analysis (PCA) with the top 100 principal components (PCs) on the normalized expression matrix. Following PCA, the appropriate PCs were selected for clustering via specific resolution parameters. Finally, cell type‐specific DEGs between WT and *Pak2^−/−^* embryos were detected using the Seurat “FindAllMarkers” function and the “MAST” method. Genes with > 0.25‐fold difference (log‐scale) on average and *p* < 0.05 were considered significant.

The DEGs from Seurat were used as input for clusterProfiler (version 3.14.3) to identify GO pathways and Kyoto Encyclopedia for Genes and Genomes (KEGG) pathways. The selected pathways were visualized using ggplot2.

To generate a trajectory, neuromesodermal progenitors, radial glia, neural progenitors, motor neuron progenitors, dorsal progenitors, diencephalon/roof plate, zona limitans intrathalamica (ZLI)/hypothalamic floor plate, floor plate/basal plate, isthmus rostral, telencephalon, and neural crest were extracted from all cells in the embryos. The Monocle (version 2.9.0) algorithm was employed by using the gene‐cell matrix with UMI counts extracted from Seurat subset. An object with the parameter negbinomial size as the expression family was created by using a new “Cell Data Set” function. After dimension reduction and cell ordering, the cell trajectory was inferred using default parameters. Then, differentially expressed genes with *q* value < 0.0001 along the pseudotime were detected, using the “‘differentialGeneTest”’ function.

The score of neural tube development in WT and *Pak2^−/−^* cells was determined by “AddModuleScore” function in Seurat. “AddModuleScore” calculated the module scores for feature expression in genes involved in neural tube development obtained from Gene Ontology biological processing pathways. The same method was used for calculating the scores for BMP signaling, forebrain and diencephalon development. We visualized the scores and the relative expression (measured by log2 (TPM + 1) in Monocle) of marker genes in the specific cell types using ggplot2.

### Coimmunoprecipitation (CoIP)

Protein extraction from cell samples was performed in NP‐40 lysis buffer (P0013F, Beyotime) with protease and phosphatase inhibitors. The extracts were preincubated for 30 min on ice. Proteins were immunoprecipitated using either a monoclonal anti‐HA antibody (1:1000, Cat^#^ 2367, Cell Signaling Technology) or monoclonal anti‐FLAG antibody (1:1000, Cat^#^ 8146, Cell Signaling Technology) at 4 °C overnight. Immunoprecipitated proteins were purified using Pierce protein A/G beads (Cat^#^ 88802, Thermo Fisher Scientific) at room temperature for 0.5 h. Finally, the proteins were eluted using NP‐40. Immunoprecipitated proteins were analyzed by western blotting using either a monoclonal anti‐FLAG antibody or monoclonal anti‐HA antibody.

### Generation of Zebrafish Pak2a Mutant Lines Using the CRISPR‐Cas9 System

The WT zebrafish line Tuebingen (Tu) was maintained, raised, crossed, and staged as previously described.^[^ ^57^ ^]^ Embryos were raised in Holtfreter's buffer at 28.5 °C. Guide RNA (gRNA) target sequences were selected using the CHOPCHOP online tool v1 (gRNA1: 5′‐GGTGCTCATTCTGACT GGGG‐3′; gRNA2: 5′‐GGGGCAGGGGTTTAGAGCTG‐3′) and generated by in vitro transcription as previously described before.^[^ ^58^ ^]^ Founder fish (F0) were developed by co‐injection of gRNAs and Cas9 protein into embryos at the 1‐cell stage. F0 embryos were raised and outcrossed against WT fish to ensure germline transmission and establish a stable line. F0 and F1 fish‐carrying mutations were identified by digestion with T7 endonuclease I, which recognizes and cleaves non‐stringently matched DNA. The obtained mutant line, *pak2a* ^Δ14^, was used for subsequent analysis.

Zebrafish embryos were fixed in paraformaldehyde (4%) in PBS for 24 h at 4 °C. The samples were then embedded in resin according to standard procedures. Transverse sections (0.5 µm) were obtained using a Leica RM2255 microtome (Wetzlar, Germany). Toluidine blue staining was performed according to standard procedures. Imaging was performed using a Nikon Eclipse Ni compound microscope (Tokyo, Japan) equipped with a Nikon camera.

Capped mRNAs were transcribed in vitro from the corresponding linearized plasmids for WT‐*PAK2*, *PAK2*‐P151S, *PAK2*‐E253A, and *PAK2*‐K278R using the mMessage mMachine kit (Ambion). The mRNAs were purified using the miRNeasy Mini Kit (Qiagen), according to the manufacturer's instructions. All mRNAs were microinjected into the yolks of one‐cell‐stage embryos.

### Statistical Analysis

Results are presented as the mean ± SEM. Statistical tests were performed using one‐way ANOVA with Dunnett's or Tukey's multiple comparisons and unpaired *t*‐tests. All fluorescence and gel images were obtained using ImageJ analysis. All of the statistical analyses were performed using GraphPad Prism software (GraphPad Software, San Diego, CA, USA).

## Conflict of Interest

The authors declare no conflict of interest.

## Supporting information

Supporting Information

Supporting Information

Supporting Information

Supporting Information

## Data Availability

For sing‐cell transcriptome sequencing and RNA‐sequencing of WT and *Pak2^−/−^* embryos at embryonic day 9.5 (E9.5), the data have been deposited to the Genome Sequence Archive (https://ngdc.cncb.ac.cn/gsa/) at the BIG Data Center, Beijing Institute of Genomics, Chinese Academy of Sciences, under the accession number PRJCA010387 (CRA007504).
