# Supplementary material for: Loss‐of‐Function of p21‐Activated Kinase 2 Links BMP Signaling to Neural Tube Patterning Defects
Source: Adv Sci (Weinh). 2022 Dec 11;10(4):2204018. doi: 10.1002/advs.202204018 (PMC9896034; doi:10.1002/advs.202204018)
Supplement: Supplementary file 1 — Supporting Information [file ADVS-10-2204018-s003.pdf]

ADVANCED SCIENCE
Open Access

Supporting Information

for *Adv. Sci.*, DOI 10.1002/advs.202204018

Loss-of-Function of p21-Activated Kinase 2 Links BMP Signaling to Neural Tube Patterning Defects

Yan Wang, Kaifan Zhang, Jin Guo, Shuyan Yang, Xiaohui Shi, Jinrong Pan, Zheng Sun, Jizhen Zou, Yi Li, Yuanyuan Li, Tianda Fan, Wei Song, Fang Cheng, Cheng Zeng, Jinchen Li, Ting Zhang and Zhong Sheng Sun**

Supporting Information

Loss-of-Function of p21-Activated Kinase 2 Links BMP Signaling to Neural Tube Patterning Defects

Yan Wang, Kaifan Zhang, Jin Guo, Shuyan Yang, Xiaohui Shi, Jinrong Pan, Zheng Sun, Jizhen Zou, Yi Li, Yuanyuan Li, Tianda Fan, Wei Song, Fang Cheng, Cheng Zeng, Jinchen Li, Ting Zhang, Zhong Sheng Sun**

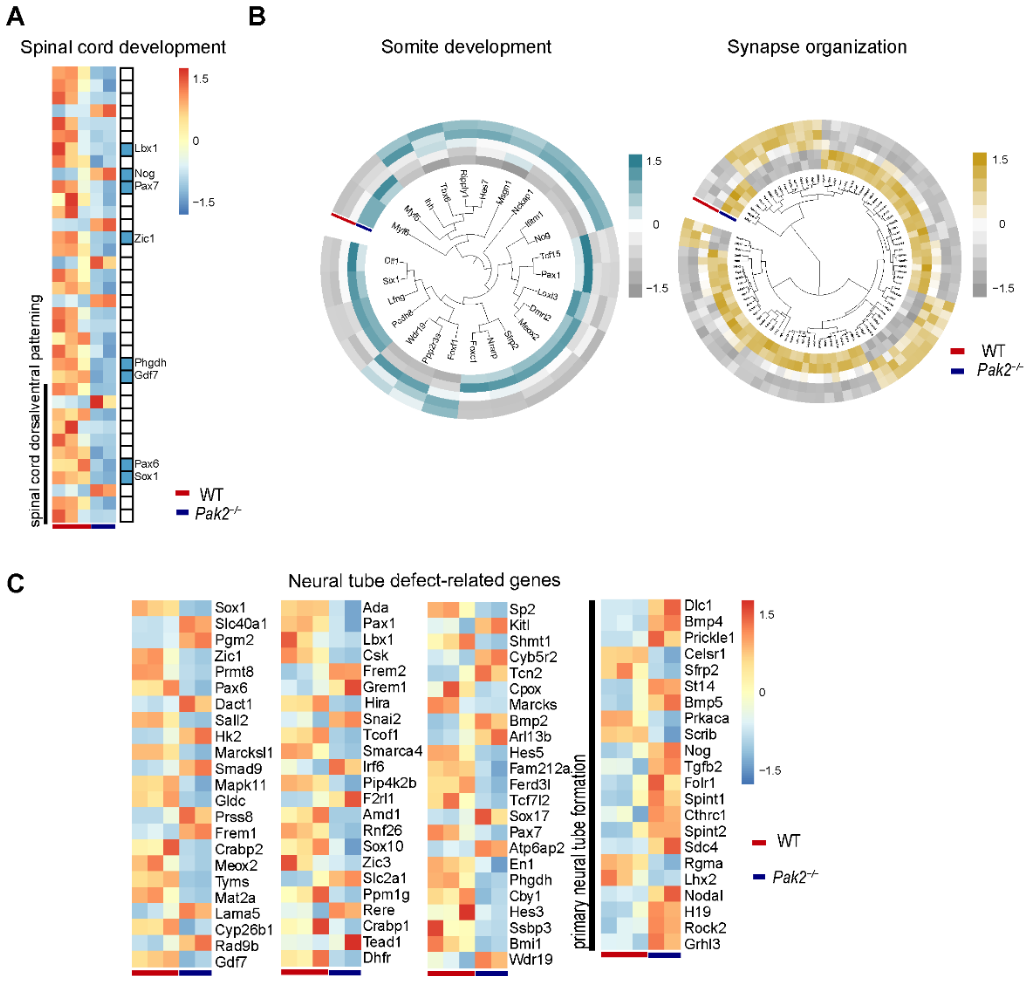

Figure S1. RNA-seq analysis of WT and *Pak2*$^{-/-}$ embryos at E9.5. A)–B) Gene ontology (GO) analysis of the DEGs in *Pak2*$^{-/-}$ bulk embryos showed the enriched functions in spinal cord development, somite development, and synapse organization at E9.5. C) Heat map showing the DEGs that were reported NTD-related genes in *Pak2*$^{-/-}$ bulk embryos at E9.5. Scale bar: TPM after log2 transforms and Z-score normalization.

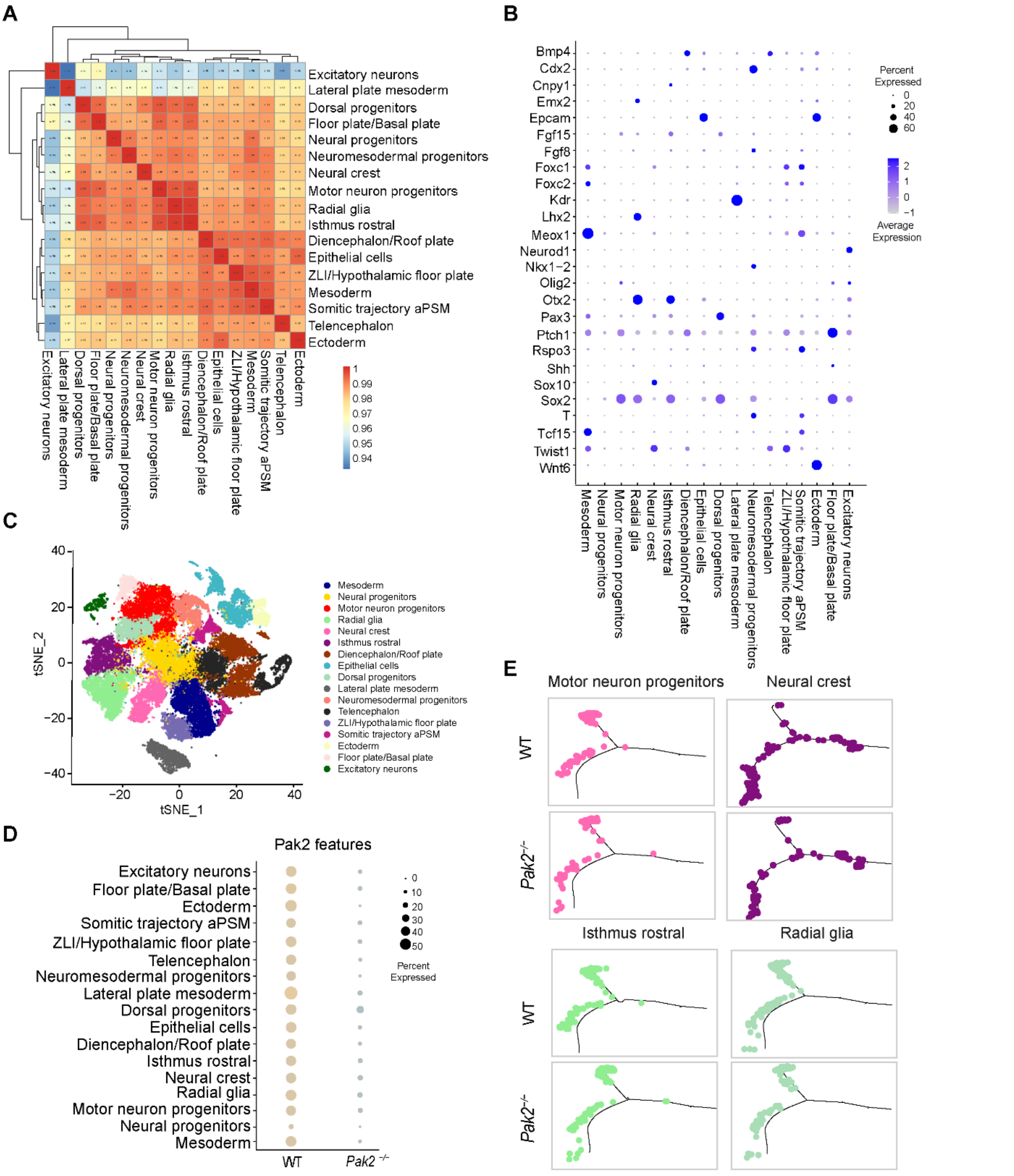

Figure S2. scRNA-seq analysis of WT and *Pak2*$^{-/-}$ embryos at E9.5. A) The correlation among the cell cluster in WT and *Pak2*$^{-/-}$ embryos. B) Heatmap of the expressions of the marker genes per cell cluster. C) T-distributed stochastic neighbor embedding (t-SNE) plots of scRNA-seq show the annotation and color codes for cell clusters in the embryos at E9.5. D) *Pak2* level in different cell cluster between WT and *Pak2*$^{-/-}$ embryos. E) Representative

developmental trajectory of cell types from WT and *Pak2*$^{-/-}$ samples via Pseudotime-ordered analysis.

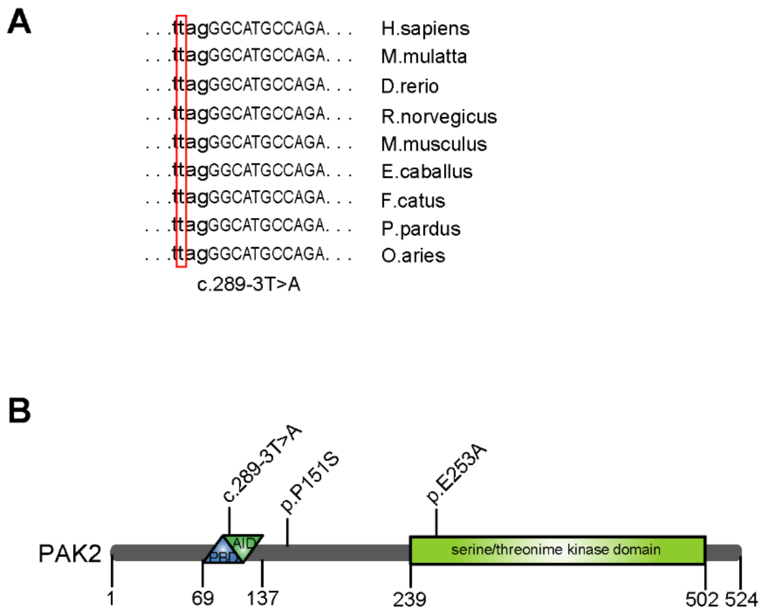

Figure S3. The information of *PAK2* rare variants in human NTD cases. A) Sequence conservation analysis of the splice site at c.289-3T of *PAK2* among multiple vertebrates. B) Schematic diagram of the full-length PAK2 protein and the locations of *PAK2* mutations in the protein.

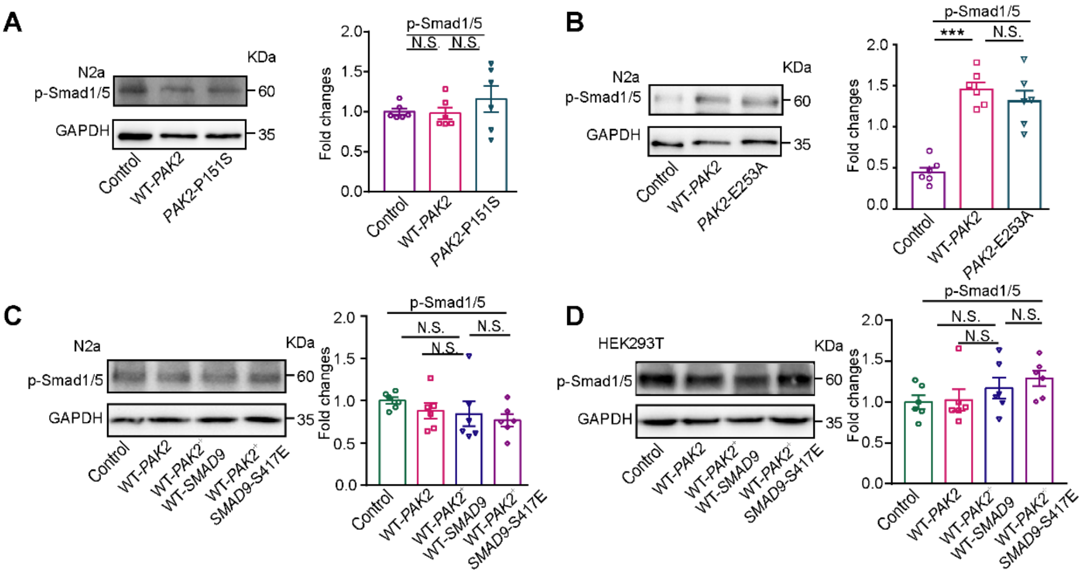

Figure S4. PAK2 did not regulate the phosphorylation levels of Smad1/5. A) The levels of p-Smad1/5 in N2a cells transfected with WT-*PAK2* and *PAK2*-P151S. $F (2, 15) = 0.858$, $p = 0.4438$, one-way ANOVA with Dunnett's multiple comparison test. B**)** The levels of p-Smad1/5 in N2a cells transfected with WT-*PAK2* and *PAK2*-E253A. $F (2, 15) = 30.43$, $p < 0.0001$; WT-*PAK2* vs *PAK2*-E253A, $p > 0.05$;for WT-*PAK2* vs Control, $p < 0.001$; one-way ANOVA with Dunnett's Multiple Comparison Test. C**)** The levels of p-Smad1/5 in N2a cells transfected with control, WT-*PAK2*, WT-*PAK2*/WT-*SMAD9*, and WT-*PAK2*/*SMAD9*-S417E plasmid. $F (2, 15) = 1.005$, $p = 0.4110$, one-way ANOVA with Dunnett's multiple comparison test. D**)** The levels of p-Smad1/5 in HEK293T cells transfected with control, WT-*PAK2*, WT-*PAK2*/WT-*SMAD9*, and WT-*PAK2*/*SMAD9*-S417E plasmid. $F (2, 15) = 1.499$, $p = 0.2454$, one-way ANOVA with Dunnett's multiple comparison test. $n = 6$ cultures for each group.

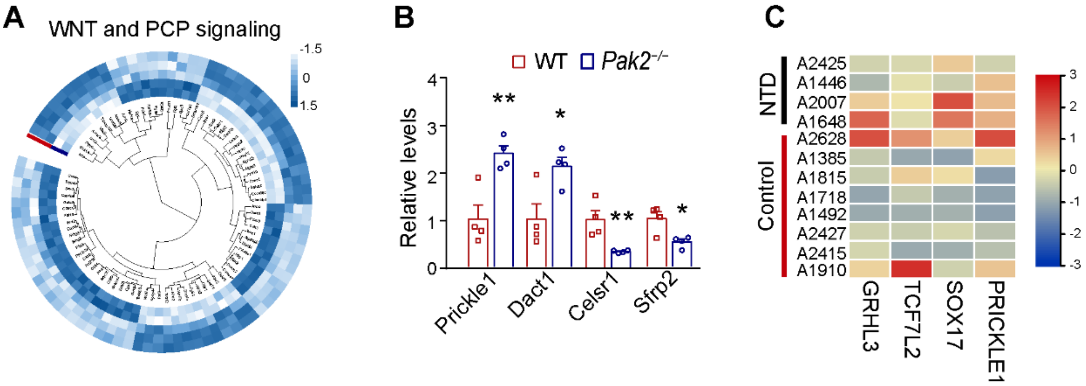

Figure S5. Abnormal WNT signaling associated with PAK2 dysfunction. A)–B) The levels of several key components in WNT signaling were altered in *Pak2*$^{-/-}$ embryos at E9.5, which was revealed via bulk RNA-seq and was verified by RT-PCR (unpaired t test. *n* = 4 embryos for each genotype). C) The levels of key components in WNT signaling in brain tissues of health controls and fetuses carrying *PAK2* mutations as shown using the NanoString nCounter RNA assay. Color bar represents log2 fold-change.
